# Medical Encounters During the 2026 AETF European Taekwon-Do Championships: A Five-Day Retrospective Descriptive Study

**DOI:** 10.3390/jcm15197544

**Published:** 2026-09-28

**Authors:** Michalina Błażkiewicz, Jacek Wąsik, Kirk Lee, Katarzyna Cupryś

**Affiliations:** 1 Faculty of Rehabilitation, The Józef Piłsudski University of Physical Education in Warsaw, 00-968 Warsaw, Poland; 2 Institute of Physical Culture Sciences, Jan Długosz University in Częstochowa, 42-200 Częstochowa, Poland; 3 AETF Anti-Doping & Health Promotion Committee, All Europe Taekwon-do Federation (AETF), 20-884 Lublin, Poland; kirkleeconsultancy@icloud.com (K.L.); k.cuprys@itfeurope.org (K.C.)

**Keywords:** Taekwon-do, sports injuries, injury surveillance, combat sports, concussion, ringside medicine, digital health, sports medicine

## Abstract

**Background/Objectives**: Injury surveillance is essential in combat sports, but recorded medical encounters may depend on assessment thresholds, competition regulations, and ringside medical organization. This study characterized medical encounters requiring assessment by the competition medical team during a five-day European Taekwon-do competition according to their number, operational severity, anatomical location, and impact on continued participation. **Methods**: A retrospective descriptive analysis was conducted based on 242 recorded medical encounters collected over five consecutive days of competition. The individual medical encounter served as the unit of analysis. Data were collected using a hybrid paper- and digital-based reporting system. Variables included competition day, location, medical-encounter category, operational severity classification, ability to continue competition, hospital referral, ambulance transport, and clinical description. **Results**: A total of 242 medical encounters were recorded: 58 on Day 1, 75 on Day 2, 18 on Day 3, 30 on Day 4, and 61 on Day 5. Most recorded medical encounters were classified as minor (239/242; 98.8%), while three were classified as moderate (1.2%). The most frequently recorded medical-encounter categories were head (83/242; 34.3%), abdomen (34/242; 14%), knee (21/242; 8.7%), and ankle (17/242; 7%). Most medical encounters were associated with continued participation (230/242; 95%). Hospital referral occurred in two cases, including one ambulance transfer. The number of recorded medical encounters decreased markedly after Day 2, coinciding with a change in the operational threshold for medical assessment of very minor problems. **Conclusions**: The number and characteristics of recorded medical encounters varied across the competition, with the observed patterns also reflecting competition procedures and reporting practices. The hybrid paper- and digital-based system provided a pragmatic approach to competition medical surveillance, while standardized definitions, consistent assessment procedures, and reliable digital infrastructure are needed. Future studies should incorporate athlete-exposure and bout-level data to enable valid exposure-adjusted injury incidence comparisons.

## 1. Introduction

Taekwon-do is a dynamic combat sport characterized by high-speed kicking techniques, rapid changes in direction, and frequent physical contact [1,2,3]. The biomechanical characteristics of Taekwon-do kicking techniques have also been investigated, including the effects of target characteristics on the velocity of roundhouse kicks [4,5]. These characteristics expose athletes to a considerable risk of acute injuries during competition and make injury surveillance an important component of athlete health protection and competition medical care [6,7,8,9]. Previous epidemiological studies have demonstrated substantial injury rates in competitive Taekwon-do, although considerable variation exists between published estimates. A meta-analysis of competition studies reported a pooled injury rate of 79.3 per 1000 athlete-exposures, with the lower extremities and contusions representing the most frequently reported injury location and type [6]. A subsequent systematic review also identified the lower extremities as the most frequently affected anatomical region, while head and neck injuries represented an important proportion of competition-related injuries [7]. More recent evidence confirms that lower-limb injuries, particularly those involving the foot and knee, remain common in high-performance Taekwon-do, although reported incidence varies according to the population, competition level, injury definition, and exposure denominator [8].

Head injuries are of particular clinical importance because of the potential consequences of concussion and traumatic brain injury. Previous research has documented head blows and concussions during competition Taekwon-do [10]. Current international consensus recommendations emphasize standardized recognition, assessment, and management of concussion in contact and collision sports [11].

Despite the growing body of epidemiological literature, direct comparison between Taekwon-do studies remains difficult. Differences in competition rules, athlete characteristics, injury definitions, severity criteria, exposure measures, and surveillance methodology may substantially influence the reported frequency and profile of injuries [6,7,12,13]. The International Olympic Committee (IOC) consensus statement therefore recommends standardized definitions and classification of health problems, consistent assessment of severity and consequences, systematic recording of athlete exposure, and transparent reporting of injury-surveillance methodology [14].

Importantly, the number of medical encounters recorded during a competition does not necessarily correspond to the number of clinically significant injuries. In this study, a medical encounter refers to an occasion on which a competitor was assessed or attended by the competition medical team and the event was entered into the surveillance system. Such encounters may represent a broad clinical spectrum, ranging from minor complaints and precautionary assessments without confirmed injury to injuries requiring removal from competition, hospital referral, or emergency transport. Consequently, the number of recorded medical encounters may depend not only on the occurrence of clinically relevant medical problems but also on the threshold for requesting or providing medical assessment, referee procedures, competition regulations, and the organization of ringside medical services. From an organizational perspective, these encounters remain relevant because they determine medical workload and may influence the allocation of medical personnel, equipment, emergency resources, and competition time.

Most previous Taekwon-do studies have focused primarily on injury incidence, injury mechanisms, anatomical distribution, or time-loss outcomes [6,7,8]. Comparatively less attention has been given to the characteristics and operational burden of recorded medical encounters during a multi-day international competition. In particular, limited information is available regarding the temporal distribution of medical encounters across consecutive competition days, their distribution across multiple competition rings, the ability of athletes to continue competing following assessment, and the need for hospital referral or ambulance transport. These factors are directly relevant to the organization and preparedness of competition medical services.

Digital injury-reporting systems may facilitate rapid data collection and improve the accessibility of surveillance data. However, their practical implementation in high-volume competition environments depends on adequate hardware, connectivity, standardized data fields, and efficient data-entry procedures. A hybrid paper- and digital-based approach may therefore represent a pragmatic solution when real-time digital access is not consistently available.

The primary outcome was the number and characteristics of recorded medical encounters requiring ringside medical attention during the 2026 AETF European Taekwon-do Championships. Characteristics included operational severity, anatomical location, competition day, competition ring, and ability to continue competition. The individual medical encounter served as the unit of analysis; therefore, the number of recorded encounters does not represent the number of unique athletes or injuries and should not be interpreted as exposure-adjusted injury incidence. A secondary outcome was subsequent medical management, including hospital referral and ambulance transport. In addition, the study evaluated the practical use of a hybrid paper- and digital-based reporting system for competition medical surveillance and explored the temporal change in the number of recorded medical encounters in relation to a change in the operational threshold for medical assessment.

## 2. Materials and Methods

### 2.1. Study Design and Setting

This study was a retrospective descriptive analysis of recorded medical encounters documented during the 2026 AETF European Taekwon-do Championships held in Maribor, Slovenia, in April 2026. The competition was conducted over five consecutive days and included five main competition rings operating according to the official competition schedule. The study was designed to characterize the medical encounters requiring assessment by the competition medical team, with particular attention to their number, anatomical location, operational severity, immediate effect on continuation of competition, and need for further medical care.

The unit of analysis was an individual medical encounter recorded in the competition medical surveillance system. A medical encounter was defined as an occasion on which a competitor was assessed or attended by the competition medical team and the event was entered into the surveillance system. The unit of analysis was therefore not necessarily a unique athlete or a unique injury. The same athlete could be assessed on more than one occasion during the competition, including for repeated complaints involving the same anatomical region. Consequently, the number of records cannot be interpreted as the number of injured athletes.

The medical service was provided by nurses and paramedics. Medical personnel were responsible for responding to calls from the competition rings, performing ringside assessments, providing immediate care, determining whether an athlete could continue, and arranging further medical evaluation or transport when required. The operational workload was substantial because medical personnel could be required to attend repeated calls from several rings simultaneously. The medical team reported that four medics could collectively be required to enter the rings hundreds of times during a competition day when five rings were operating.

### 2.2. Medical Data Collection

Medical data were collected using a hybrid paper- and digital-based reporting system. Paper-based documentation was the traditional method used before implementation of the digital application. During the 2026 European Championships, the two methods were used concurrently because the number of available iPads was insufficient to provide a device for all medical personnel working at different locations. When a device was available, medical personnel entered the encounter directly into the digital application. When a device was not available, the encounter was initially documented on paper and subsequently entered into the digital system using a computer.

The reporting approach was developed to minimize the time required for documentation during active competition. The digital system was intended to permit routine encounters to be recorded through approximately three to four rapid selections, including the event or injury category and whether the athlete continued competition. More clinically complex encounters, including suspected concussion, fracture, spinal injury, or events requiring ambulance communication, required more detailed clinical documentation. The final dataset comprised all medical records corresponding to recorded medical encounters available for the five competition days in Maribor.

### 2.3. Digital Reporting Application

The digital reporting application was developed specifically to facilitate rapid and structured recording of medical encounter data during the competition. The application enabled direct data entry and subsequent export of the collected records for analysis. At the time of the competition, the system was cloud-based and did not provide offline functionality with subsequent synchronization.

The application reproduced the principal information collected using the paper-based system. It allowed users to select the competition, competition location, country, event or injury category, severity, continuation status, and other relevant information. Previously entered records could be corrected when an incorrect category had been selected. The system also generated reports and allowed rapid export of the accumulated data in CSV format for subsequent spreadsheet-based analysis.

The digital system was designed to support anonymized surveillance. Country and competition-ring information could be retained for analysis, whereas competitor names were not routinely included in the dataset used for the present study. The system had the technical capacity to use an athlete number for longitudinal tracking, but athlete-level identification was not routinely used in the analyzed Maribor dataset because of data-protection considerations.

### 2.4. Competition Location and Ring Allocation

Five main competition rings were used during the championships. Ring assignments were determined by the competition draw and schedule, with specific competition categories allocated to individual rings. During the Maribor competition, the numbering of the main rings remained stable throughout the event.

The reporting application was designed to be reusable in larger competitions and therefore contained additional ring-number options and categories such as self-presented cases and other medical encounters. These additional options reflected the flexibility of the software rather than the actual number of continuously operating rings at the Maribor championships. The analysis of ring distribution was therefore based on the competition structure actually used in Maribor.

Self-presented medical encounters were retained as a separate category because athletes could present directly to the medical team after completing or leaving competition, including cases in which a condition became apparent after the bout.

### 2.5. Recorded Variables

The surveillance system recorded information regarding the competition setting, anatomical/event category, severity, immediate functional outcome, and subsequent medical management. Variables available for analysis included competition day, competition date, competition ring or location, country, medical-encounter category, severity, ability to continue competition, inability to continue competition, hospital referral, ambulance transport, and clinical description.

The anatomical classification used broad categories based on International Olympic Committee (IOC) injury-surveillance categories, including arm, elbow, shoulder, hand, neck, head, thoracic spine, lumbar region, abdomen, hip, thigh, knee, leg, ankle, and foot. Additional categories were incorporated specifically for the purposes of this surveillance system, including concussion assessed, confirmed concussion, unspecified region, and multiple regions. The use of additional concussion-related categories reflected the need to identify not only head impacts but also cases in which a formal concussion assessment was undertaken or concussion was confirmed.

The system also permitted a free-text clinical description. The amount of narrative information varied according to the clinical significance of the encounter. For very minor events, no additional description was sometimes entered when the assessment identified no clinically significant injury and the athlete rapidly resumed participation. An empty description therefore did not necessarily indicate that no medical assessment had occurred.

### 2.6. Definition of Medical Encounter and Injury Classification

Because the surveillance system was intended to document medical activity at the competition rings, recorded medical encounters covered a broad clinical spectrum. Some encounters represented confirmed injuries, whereas others represented precautionary or very minor assessments in which no clinically significant injury was identified.

This distinction was particularly relevant during the initial phase of the competition. On the first competition day, centre referees had been instructed to request medical assistance whenever an athlete appeared to have even a minor problem requiring medical assessment. Consequently, the medical team attended some medical encounters that, after assessment, were considered very minor and did not require treatment or interruption of participation.

Accordingly, the primary terminology used throughout this study is “medical encounter.” The term “medical event” is used only when referring to the terminology of the reporting system or to specific event categories recorded in the surveillance data. The term “injury” is used when referring to the injury classification recorded by the medical team or to a clinically documented injury.

### 2.7. Severity Classification

Recorded medical encounters were classified according to the operational severity categories used by the competition medical team: minor, moderate, or severe.

Encounters classified as minor constituted the majority of recorded encounters. In general, a minor classification was assigned when the athlete could continue competition after the assessment. Examples included transient effects of a kick or punch, such as temporary abdominal discomfort or being briefly winded, when the athlete recovered and continued participation.

Severity was not, however, considered synonymous with ability to continue. The medical team specifically indicated that an encounter classified as minor could occasionally prevent continuation, for example in the case of an ankle sprain. Conversely, some athletes with minor complaints could resume competition almost immediately. Therefore, severity and continuation status were recorded and analyzed as separate variables.

Encounters classified as moderate generally represented more clinically significant conditions and were more commonly associated with inability to continue competition and/or the need for additional medical evaluation. Hospital assessment or ambulance transport could occur in this category. Encounters classified as severe represented the highest level of clinical concern within the operational classification used by the medical team. No recorded medical encounter in the analyzed dataset was classified as severe.

The operational classification was retained as recorded by the medical team and was not retrospectively converted to a standardized time-loss or validated injury-severity scale.

### 2.8. Ability to Continue Competition

Ability to continue competition was recorded separately from the severity classification. The reporting system contained fields indicating whether the athlete was able to continue and whether the athlete was unable to continue.

These variables were treated as indicators of the immediate functional consequences of the medical encounter. They were not used as a substitute for severity classification because the medical team reported that an encounter classified as minor could occasionally prevent continuation, whereas some minor complaints allowed almost immediate return to competition.

Hospital referral and ambulance transport were also analyzed separately as indicators of the subsequent level of medical care required.

### 2.9. Head Injury and Concussion Assessment

Head-related medical encounters were recorded using separate categories for head injury, concussion assessment, and confirmed concussion. This distinction allowed the surveillance system to differentiate between routine head impacts and cases in which a formal concussion assessment was performed or concussion was considered confirmed.

For clinically significant head injuries, the medical team recorded more detailed clinical information than for routine minor encounters. The documented assessment could include the mechanism of injury, loss of consciousness, neurological status, speech, memory, eye movements, and Glasgow Coma Scale assessment.

One clinically significant concussion involved loss of consciousness following a head impact and an unprotected fall. The athlete initially showed no movement, followed by altered responsiveness, transient inability to speak, abnormal eye movements, confusion, and lack of recollection of the event. Manual inline stabilization was performed during the initial assessment. Approximately five minutes after the incident, the athlete had a GCS score of 15 and was transferred for further assessment in the medical room and subsequently transported by ambulance to an emergency department. The medical team reported that clinically significant concussion cases received detailed assessment and documentation because subsequent medical care could depend on the information recorded at the time of the medical encounter.

The present study did not retrospectively establish new diagnoses. Concussion-related records were analyzed according to the classification and clinical documentation available in the competition medical surveillance records.

### 2.10. Temporal Data and Data-Quality Considerations

Competition day and date were used as the primary temporal variables. The exact time of the medical encounter was not considered sufficiently reliable for analysis.

This limitation resulted directly from the hybrid reporting process. The digital application was designed to generate a timestamp corresponding to the time of entry. However, when medical personnel initially documented an encounter on paper and later entered it into the application, the recorded timestamp represented the later digital entry rather than the actual time of the medical encounter. In busy periods, multiple paper records could therefore be entered retrospectively at the end of the day and appear to cluster around the same time. The competition date remained correct, but the exact timestamp could not reliably be interpreted as the time of injury or medical assessment.

Consequently, the analysis was performed by competition day rather than by exact time of occurrence. No minute-by-minute or hourly analysis was undertaken.

### 2.11. Changes in Medical-Call Threshold During the Competition

The interpretation of the temporal distribution of medical encounters required consideration of an operational change in the threshold for requesting medical assistance.

During the first two competition days, centre referees were instructed to call the ringside medical team whenever an athlete appeared to have even a minor problem requiring medical assessment. The resulting workload included numerous assessments in which no clinically significant injury was identified.

At the end of Day 2, the practical threshold for requesting medical assistance was changed. For clearly minor incidents, centre referees were no longer routinely required to call the medical team and could allow the athlete a short period to recover and continue. The medical team described this as a “gentleman’s agreement” between the medical personnel and referees rather than an official modification of the competition rules. The change was introduced because the first two competition days had experienced substantial delays, with the competition extending approximately two to two and a half hours beyond the anticipated finishing time.

Following this operational change, the number of recorded medical encounters decreased markedly. Day 2 included 75 recorded encounters, whereas Day 3 included 18. The medical team considered the Day 3 recorded encounters more likely to represent cases that actually required medical attention, including epistaxis and lower-limb, foot, and toe complaints.

This change was therefore treated as an important surveillance-context variable. The reduction in recorded encounters after Day 2 was not interpreted as evidence of an equivalent reduction in the underlying occurrence of injuries because the threshold for medical assessment and documentation had changed.

### 2.12. Competition Rule Context

A separate procedural change affecting competition time was introduced for headshots. According to the competition medical team, the rule requiring the competition clock to stop after a headshot had been communicated approximately one month before the European Championships. Under the new procedure, the clock stopped when the referee made the relevant headshot call. According to the medical team, this increased the amount of effective sparring time compared with the previous practice, in which time continued to run during such interruptions.

The medical team considered this change relevant to the physical demands of the competition because athletes were exposed to longer periods of active sparring. This information was treated as contextual information when interpreting the distribution of medical encounters and was not used as an individual-level exposure variable.

The final competition day consisted of team sparring, with teams of five athletes. The medical team noted that substantial differences in body size could occur between opposing athletes despite differences in the degree of physical contact permitted within the discipline. The number of medical encounters increased again during this final day.

### 2.13. Statistical Analysis

The primary outcome was the number and characteristics of recorded medical encounters. The dataset was analyzed using descriptive statistics. Categorical variables were summarized as absolute frequencies and percentages. The total number of medical encounters was calculated for the five-day competition and separately for each competition day.

The distribution of recorded medical encounters was described according to medical-encounter category, competition ring, severity, ability to continue competition, hospital referral, and ambulance transport. Clinical descriptions were reviewed descriptively to characterize selected clinically significant encounters, particularly concussion and medical encounters requiring hospital or ambulance assessment.

The analysis did not attempt to calculate athlete-level recurrence because individual athlete identifiers were not consistently available in the analyzed dataset. Consequently, repeated encounters involving the same athlete could not be reliably linked and were treated as separate medical encounters. This limitation prevents estimation of the proportion of athletes affected or the number of encounters per individual athlete.

No exposure-adjusted injury incidence rates were calculated in the primary analysis because the medical surveillance dataset itself did not provide a reliable athlete-exposure denominator. Accordingly, the present study reports the number and characteristics of recorded medical encounters rather than injury incidence per athlete-exposure.

### 2.14. Data Protection and Ethical Considerations

The dataset provided for analysis contained no personally identifiable information. Athlete names and other direct identifiers were not included in the dataset used for the present study.

### 2.15. AI Statement

During the preparation of this manuscript/study, the authors used ChatGPT (GPT-5.6 Luna) for the purposes of language editing, improving the clarity of the text and generating Figure 2 based on the authors’ data. The authors have reviewed and edited the output and take full responsibility for the content of this publication.

## 3. Results

### 3.1. Overall Number of Recorded Medical Encounters

A total of 242 medical encounters were recorded during the five-day championship. The distribution of recorded medical encounters across competition days was uneven, with the highest number recorded on Day 2 and the lowest on Day 3. The number of recorded medical encounters subsequently increased during the final two competition days (Table 1).

### 3.2. Recorded Medical-Encounter Categories

The distribution of recorded medical-encounter categories is presented in Figure 1. The head category was the most frequently recorded medical-encounter category, followed by the abdomen, knee, and ankle. The remaining categories were individually less common and represented smaller proportions of the total number of medical encounters. Four records contained no anatomical data, while three were classified as involving an unspecified or multiple region. Concussion was recorded as confirmed in three cases and as assessed in one additional case.

### 3.3. Severity of Recorded Medical Encounters

Of the 242 recorded medical encounters, 239 (98.8%) were classified as minor, while three (1.2%) were classified as moderate. No recorded medical encounters were classified as severe (Table 2). The three medical encounters classified as moderate occurred on Days 4 and 5. Two moderate encounters occurred on Day 4. One involved a right abdominal/flank injury following a kick to the kidney region, accompanied by vomiting. The second involved a head injury following a kick to the head. The third medical encounter classified as moderate occurred on Day 5 and involved a confirmed concussion associated with loss of consciousness and subsequent ambulance transport.

### 3.4. Participation and Medical Management Following Medical Assessment

Following medical assessment, the large majority of medical encounters were associated with continued participation, whereas only a small proportion were associated with inability to continue (Table 3). The separate variable indicating inability to continue was recorded as “Yes” in nine cases (3.7%). Three records showed an inconsistency between the two continuation-related variables, with “No” recorded in both fields. Hospital referral was uncommon, being documented in two cases (0.8%), while one medical encounter (0.4%) involved ambulance transport to hospital (Table 3).

Four records contained concussion-related classifications. Three records were classified as confirmed concussion, while one record was classified as concussion assessed. Of the three confirmed concussion records, two were classified as minor and one as moderate.

The moderate concussion occurred on Day 5 and was associated with loss of consciousness following a head impact and an unprotected fall. The clinical record additionally documented an initial period without movement, transient aphasia, confusion, impaired recall, and abnormal eye movements. A Glasgow Coma Scale score of 15 was subsequently recorded, followed by removal from the competition area and ambulance transport to hospital. This was the only concussion-related medical encounter associated with both loss of consciousness and ambulance transport.

The four concussion-related records represented 1.7% of all recorded medical encounters. Overall, most medical encounters did not prevent continued participation, while hospital referral and ambulance transport were uncommon.

### 3.5. Spatial and Temporal Distribution of Medical Encounters

The spatial distribution of recorded medical encounters across the competition venue showed the highest numbers at the principal competition rings (Figure 2). The largest number of recorded medical encounters was at Ring 1, followed by Ring 3 and Ring 5, while smaller numbers were recorded at Ring 2 and Ring 4. Only a small proportion of encounters were classified as Self Presented, Ring 6, Other, or Power Technique.

The four records assigned to Ring 6 were retained as recorded in the dataset. Because the reporting application included additional ring-location options beyond the principal competition rings, these records should not be interpreted as evidence of continuous operation of a sixth principal competition ring.

The temporal distribution of medical encounters varied substantially across the five competition days (Figure 3). The highest number of recorded medical encounters was recorded on Day 2, whereas the lowest number of recorded medical encounters was recorded on Day 3. A marked reduction occurred between these two days, with the number of recorded encounters decreasing from 75 to 18, corresponding to a 76% decrease.

This reduction coincided with an adjustment in the practical threshold for requesting medical assessment. During the first two competition days, referees routinely requested medical assistance also for very minor complaints. At the end of Day 2, the medical team and referees agreed to modify this operational approach, and very minor complaints were no longer routinely referred for formal medical assessment. The change was introduced in the context of substantial competition delays. Following this adjustment, the encounters recorded on Day 3 were considered by the medical team to be more likely to represent cases requiring medical attention.

The number of encounters increased again on Days 4 and 5. Day 5, which included team sparring, accounted for 61 medical encounters and represented the second-highest daily number recorded during the championship. Team sparring involved teams of five athletes, and differences in body size between opposing competitors could occur within this format. The medical encounters recorded on Day 5 were predominantly classified as minor, with one moderate abdominal/flank encounter and one confirmed concussion requiring ambulance transport.

The available data do not allow the independent contribution of competition format, athlete exposure, or changes in the threshold for requesting medical assessment to the observed temporal variation to be quantified.

## 4. Discussion

### 4.1. Anatomical Distribution and Comparison with Previous Taekwon-Do Literature

The present study identified 242 medical encounters recorded during a five-day European Taekwon-do championship. The most frequently recorded anatomical category was the head region, accounting for 83 encounters (34.3%), followed by the abdomen (14%), knee (8.7%), and ankle (7%). This distribution differs from the pattern commonly reported in epidemiological studies of competitive Taekwon-do, in which lower-extremity injuries have generally predominated. Previous reviews have reported that approximately 45–53% of competition injuries involve the lower extremities [15], whereas head and neck injuries account for a smaller but clinically important proportion of cases [8,16,17]. Recent prospective surveillance data from the 2019 World Taekwon-do Championships similarly demonstrated a predominance of lower-extremity injuries, while also highlighting the influence of competition rules and surveillance methodology on observed injury patterns [18].

Several methodological differences should be considered when comparing these findings. First, the present study analysed medical encounters recorded by the competition medical team rather than exposure-adjusted injury incidence. Second, the anatomical category “Head” included a broad group of head-related medical encounters and should not be interpreted as equivalent to diagnosed concussion or traumatic brain injury. Only three encounters were classified as confirmed concussion, with one additional encounter recorded as concussion assessed. Consequently, the relatively high proportion of head-related records should not be interpreted as evidence of a correspondingly high concussion incidence.

The difference between the present anatomical distribution and previous Taekwon-do literature may also reflect differences in surveillance methodology, case definitions, competition characteristics, and thresholds for medical assessment. The 2025 systematic review of high-performance Taekwon-do similarly highlighted substantial heterogeneity between studies, including differences in injury definitions and exposure denominators, and reported that lower-limb injuries, particularly foot and knee injuries, were most frequently observed in the available literature [8]. These methodological differences underline the importance of standardized definitions and reporting procedures when comparing injury data between competitions. The IOC consensus statement on sports injury and illness surveillance specifically emphasizes consistent definitions, severity classification, exposure measurement, and reporting methodology to improve comparability between studies [14].

### 4.2. Severity of Recorded Medical Encounters and Immediate Competition Consequences

The majority of recorded medical encounters in the present dataset were classified as minor (239/242; 98.8%), while three encounters (1.2%) were classified as moderate and none as severe. In parallel, 230 encounters (95%) were recorded as associated with the athlete being able to continue participation. These findings indicate that most recorded medical encounters were not associated with immediate cessation of participation.

The high proportion of encounters classified as minor is broadly consistent with previous research reporting a predominance of relatively low-severity injuries in high-performance Taekwon-do. The recent systematic review by García Blanco et al. [8] reported that most injuries in high-performance Taekwon-do were of low severity, although definitions of severity and time loss differed between studies [19]. However, the present findings should not be directly interpreted using time-loss concepts because severity was based on the operational classification recorded during the competition rather than retrospectively converted to a standardized time-loss definition.

Similarly, the ability to continue participation should not be considered synonymous with low clinical importance. An athlete may continue competing after a medical assessment despite the presence of a condition requiring subsequent observation or treatment. Conversely, a medical encounter may represent an assessment of a relatively minor complaint rather than a clinically significant injury. The distinction between medical encounters, injuries, and health problems is therefore important when interpreting the present dataset and is consistent with the broader IOC recommendations for sports injury surveillance [14].

### 4.3. Concussion and Medical Preparedness

Although confirmed concussion represented only three records in the dataset, head-related medical encounters were the most frequent anatomical category. One clinically significant case involved loss of consciousness following an unprotected fall, accompanied by transient neurological symptoms and subsequent ambulance transfer to an emergency department. Two additional cases were classified as confirmed concussion without ambulance transport. These cases illustrate that the overall predominance of minor encounters does not eliminate the need for appropriate ringside preparedness for potentially serious neurological events.

The contemporary international consensus on sport-related concussion emphasizes prompt recognition, appropriate clinical assessment, and individualized management of suspected concussion. The Amsterdam 2022 consensus also stresses the need for evidence-informed assessment tools and appropriate medical decision-making following suspected sport-related concussion [11,20,21].

The present dataset cannot be used to estimate concussion incidence because the necessary athlete-exposure denominator was unavailable. Nevertheless, the occurrence of clinically significant head trauma during the championship demonstrates the operational importance of having appropriately trained medical personnel and emergency referral pathways available during combat-sport competition. Previous Taekwon-do research has also identified head impacts and concussion as important components of competition-related medical surveillance [16].

### 4.4. Temporal Variation and Influence of Competition Procedures

The number of recorded medical encounters varied substantially across the five competition days. The highest number was recorded on Day 2 (75 encounters), whereas only 18 encounters were recorded on Day 3. This represents a 76% reduction in the number of recorded medical encounters between the two days. Importantly, this change coincided with an operational change in the threshold for requesting medical assessment. During the first two days, referees were encouraged to call medical personnel even for relatively minor complaints, whereas from Day 3 onward the practical threshold for requesting medical assistance was modified.

Therefore, the reduction in recorded encounters between Days 2 and 3 should not be interpreted as evidence of a corresponding reduction in the underlying occurrence of injuries or medical problems. Rather, the observation demonstrates how surveillance data can be affected by changes in case ascertainment and reporting procedures. This is particularly relevant in competition-based surveillance, where medical personnel, referees, athletes, and event procedures collectively determine which events are documented.

This observation is consistent with the general principles of sports injury surveillance, in which changes in case definitions, reporting procedures, or data collection methods may influence observed injury patterns. The IOC consensus recommendations emphasize the importance of clearly defining the health problem being monitored and maintaining consistency in data collection procedures when comparing injury patterns over time [14].

A separate procedural change concerned the introduction of a headshot-related clock-stop rule. According to the competition procedures, the rule had been communicated before the event and resulted in changes to the effective duration of sparring. Although the present study cannot quantify the independent effect of this rule on medical-encounter occurrence, its presence represents an additional competition-level factor that should be considered when interpreting temporal patterns.

### 4.5. Spatial Distribution and Competition Medical Workload

Medical encounters were also distributed unevenly across the competition venue. Ring 1 accounted for 65 records (26.9%), followed by Ring 3 with 59 (24.4%) and Ring 5 with 50 (20.7%). These data provide a description of the workload experienced by the medical team at different locations within the competition venue.

However, these numbers cannot be interpreted as evidence that some rings were inherently more hazardous than others. The number of medical encounters at a given ring depends on multiple factors, including the number and duration of bouts, athlete traffic, competition schedule, ring assignment, and the probability that an event was reported. Because the present study did not include reliable bout-level exposure data for each ring, exposure-adjusted rates of injury or medical encounters per bout or athlete-exposure could not be calculated [22].

The spatial data nevertheless have practical value for event organization. They demonstrate that medical surveillance can generate information not only about the clinical characteristics of encounters but also about the distribution of medical workload across a competition venue. Future studies incorporating the number and duration of bouts at each ring could determine whether observed differences in medical encounters correspond to differences in exposure-adjusted risk.

### 4.6. Team Sparring and Competition Format

The final competition day consisted of team sparring and was associated with 61 recorded medical encounters, representing 25.2% of the total dataset. The team format differed from the preceding individual competition days, including differences in bout organization and team composition. The higher number of recorded encounters on the final day may therefore be of operational interest.

Nevertheless, the present study cannot establish that team sparring itself caused a higher number of recorded medical encounters. The final day also differed from earlier days in several other respects, including competition schedule, accumulated exposure, participant fatigue, operational procedures, and the number and organization of bouts [23]. Without bout-level exposure data and appropriate comparison groups, the independent contribution of team sparring cannot be determined.

Previous research has identified competition characteristics and athlete-related factors as potential contributors to injury occurrence in Taekwon-do [24]. For example, a systematic review of risk factors reported an association between heavier weight divisions and higher overall injury incidence, while emphasizing that relatively few potential risk factors had been prospectively investigated [6,25]. This supports the need for future studies to collect more detailed exposure and competition-level information when examining the influence of competition format.

### 4.7. Hybrid Digital–Paper Surveillance

An additional finding of practical relevance was the use of a hybrid paper- and digital-based reporting system. Medical records were entered directly into the reporting application when appropriate devices were available, whereas paper-based documentation was used when digital entry was not feasible and was subsequently transcribed. Web-based injury surveillance has also been used in Taekwon-do research. Son et al. [26] implemented a web-based injury surveillance system to prospectively monitor injuries among Korean elite Taekwon-do athletes. This approach provided a structured method for collecting injury surveillance data during competition. At the same time, the need for transcription introduces an additional stage in the data pathway and may increase the possibility of missing or inconsistent information. The dataset also demonstrated variability in the completeness of free-text descriptions. Importantly, an incomplete narrative description does not necessarily indicate that no clinical assessment was performed.

The experience of this competition supports the practical importance of designing digital surveillance systems around the conditions in which medical teams actually work. In particular, systems intended for ringside or field-based use should provide reliable operation when connectivity or device availability is limited. However, the present study did not formally compare digital and paper-based data collection in terms of accuracy, completeness, or usability. Therefore, no conclusion regarding the superiority of either method can be drawn from the present data.

From a research perspective, standardized digital reporting could facilitate more consistent data collection and subsequent analysis, particularly if predefined fields for injury definition, anatomical location, severity, participation status, and follow-up are incorporated. Such standardization would be consistent with the IOC recommendations for sports injury surveillance and reporting [14].

### 4.8. Study Limitations

Several limitations should be considered when interpreting the findings. First, the study was based on a single five-day European championship and therefore describes one specific competition environment. The findings cannot automatically be generalized to other Taekwon-do competitions, age groups, competition levels, or organizational settings.

Second, the unit of analysis was a medical encounter rather than a unique athlete or a unique injury. The same athlete could therefore potentially contribute more than one record. Because athlete-level identifiers were not available for analytical use, recurrent encounters involving the same athlete could not be reliably identified.

Third, no reliable athlete-exposure denominator was available. Consequently, the study cannot provide injury incidence rates per athlete-exposure, bout, minute of competition, or other exposure-based measure. The frequencies reported for individual competition days and rings should therefore be interpreted as counts of recorded medical encounters rather than measures of injury risk.

Fourth, the threshold for requesting medical assessment changed during the competition. This procedural change represents an important potential source of variation in case ascertainment and limits direct comparison of encounter counts between competition days.

Fifth, data were collected using both digital and paper-based methods. Paper records subsequently required transcription, creating a potential source of information loss or inconsistency. In addition, some free-text descriptions were incomplete, limiting the level of clinical detail available for selected records. The hybrid reporting procedure also limited the reliability and comparability of recorded timestamps, particularly for encounters initially documented on paper and entered into the digital system retrospectively.

Sixth, the operational severity classification used during the competition was not retrospectively converted into a validated time-loss or standardized injury-severity scale. The classification should therefore be understood as a description of the medical team’s recorded assessment rather than as a standardized epidemiological severity measure.

Finally, the study did not contain sufficient bout-level information to separate the effects of competition day, competition format, rule-related changes, athlete exposure, and other contextual factors. Accordingly, the observed temporal and spatial differences should be interpreted descriptively and should not be considered evidence of causal relationships.

Despite these limitations, the dataset provides a detailed description of medical encounters recorded during a major European Taekwon-do championship and illustrates several methodological factors that are important when interpreting competition-based medical surveillance data. Future studies should combine standardized injury definitions with athlete-exposure and bout-level data, stable reporting procedures, and reliable digital systems to improve the comparability and epidemiological value of Taekwon-do injury surveillance.

## 5. Conclusions

A total of 242 medical encounters were recorded during the five-day 2026 European Taekwon-do Championships, with the vast majority classified as minor and associated with continued participation. Head-related medical encounters represented the most frequently recorded anatomical category, whereas hospital referral and ambulance transport were uncommon. The findings also demonstrated substantial temporal variation in recorded medical encounters. In particular, the marked decrease between Days 2 and 3 coincided with a change in the operational threshold for medical assessment, indicating that surveillance counts may vary not only according to the occurrence of medical encounters but also according to competition procedures and reporting practices.

The observed distribution of medical encounters across competition rings provides information on the medical team’s operational workload; however, without athlete-exposure or bout-level data, these findings cannot be interpreted as differences in injury risk between locations or competition days. Similarly, the higher number of encounters recorded during the final team-sparring day should be interpreted descriptively rather than as evidence of a causal effect of competition format.

The hybrid paper- and digital-based reporting system enabled systematic collection of medical records during a large international competition, but the experience also highlighted the importance of consistent assessment thresholds, standardized definitions, reliable digital infrastructure, and offline-capable reporting systems. Future multi-event studies should incorporate athlete-exposure data, bout-level information, standardized injury and concussion definitions, and consistent reporting procedures to allow valid comparison of medical-encounter patterns and exposure-adjusted injury incidence across competitions.

Overall, these findings provide a descriptive account of medical workload during a five-day European Taekwon-do championship and highlight the importance of considering medical encounters, competition format, and operational procedures when interpreting competition-based medical surveillance data.

## Figures and Tables

**Figure 1:**
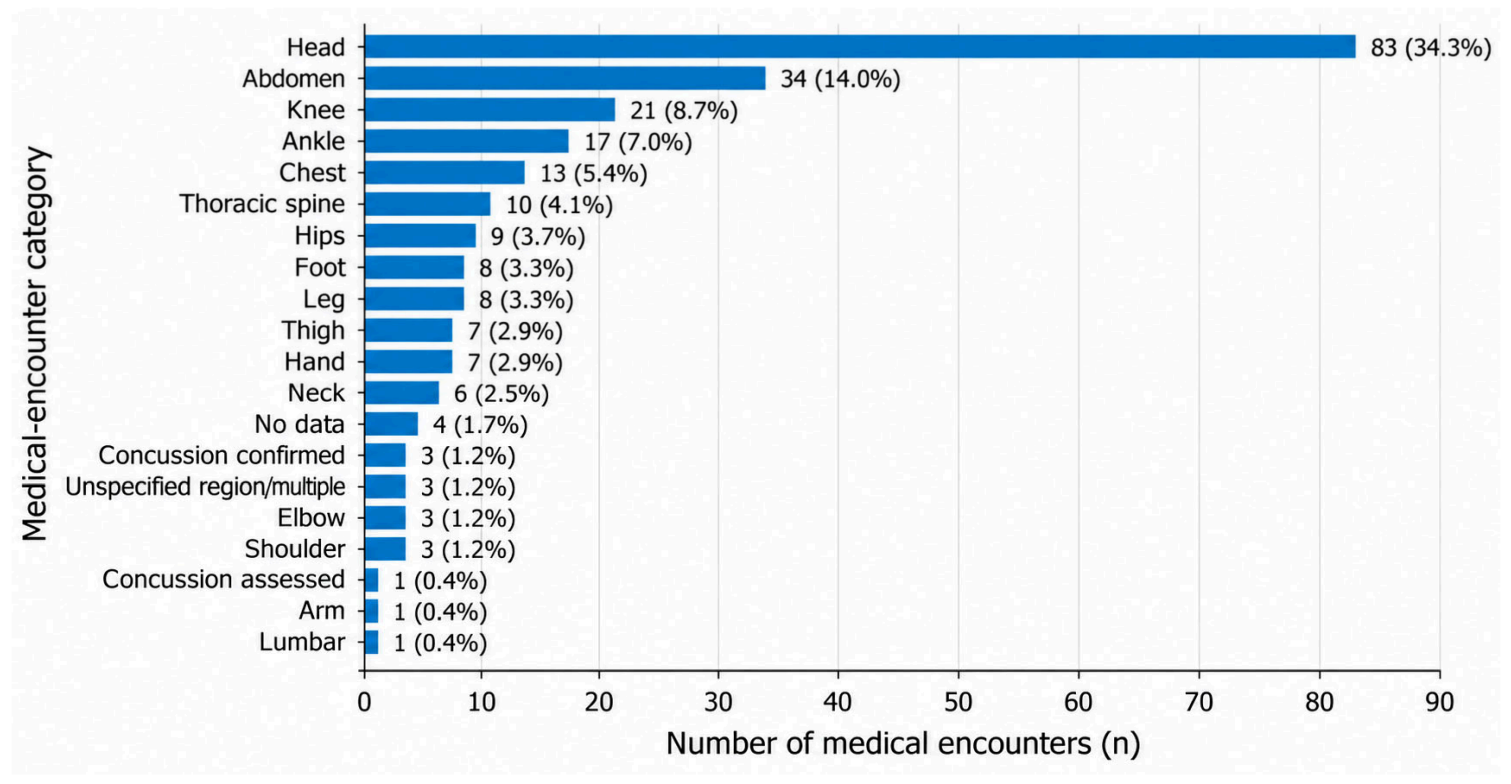
Distribution of recorded medical-encounter categories. Number of recorded medical encounters according to the recorded category. Values represent the number of encounters (n) and percentage of all recorded encounters (N = 242).

**Figure 2:**
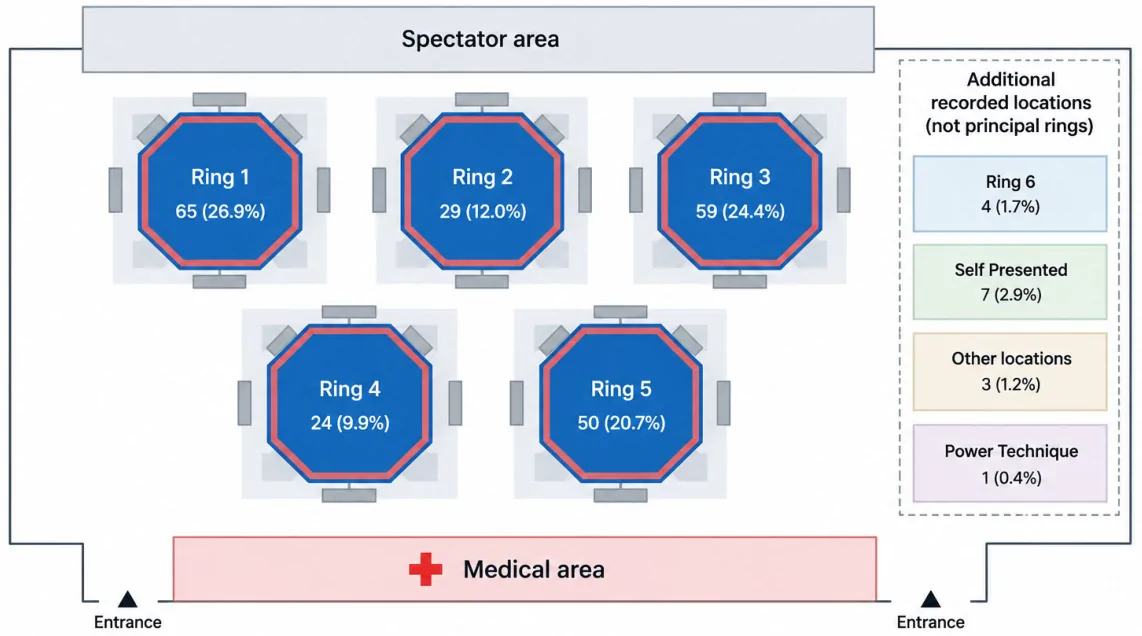
Spatial distribution of recorded medical encounters across the competition venue. Numbers indicate the number of recorded medical encounters, with percentages of the total (N = 242). Five main competition rings were used. Additional locations (including Ring 6) reflect available options in the reporting application and do not indicate an additional principal competition ring.

**Figure 3:**
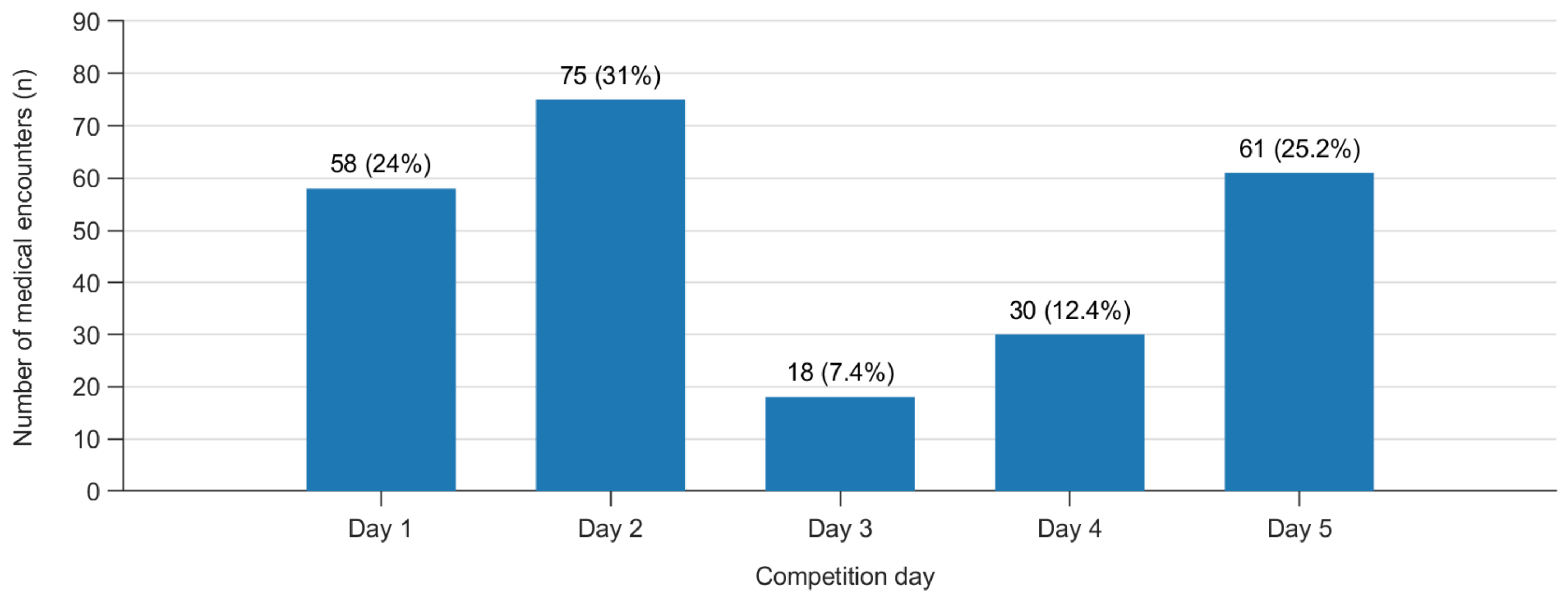
Temporal distribution of recorded medical encounters across the five competition days.

**Table 1:** Distribution of recorded medical encounters across competition days.

Competition Day	n	%
Day 1	58	24
Day 2	75	31
Day 3	18	7.4
Day 4	30	12.4
Day 5	61	25.2
Total	242	100

**Table 2:** Distribution of recorded medical encounters according to operational severity classification.

Severity	n	%
Minor	239	98.8
Moderate	3	1.2
Severe	0	0
Total	242	100

**Table 3:** Participation and medical management following medical assessment.

Outcome Following Medical Assessment	n	%
Able to continue competition	230	95
Unable to continue competition	12	5
Hospital referral	2	0.8
No hospital referral	240	99.2
Ambulance transport	1	0.4
No ambulance transport	241	99.6

Note: Participation status, hospital referral, and ambulance transport were recorded as separate variables and are therefore not mutually exclusive across domains. “Able to continue” and “Unable to continue” were recorded as separate fields and were not treated as logical complements. In three records, both fields were recorded as “No”.

## Data Availability

The data presented in this study are not publicly available due to privacy and data protection considerations concerning medical records collected during a sporting event. The aggregated data supporting the findings of this study are available from the corresponding author upon reasonable request and subject to applicable data protection requirements.
